# Prevalence and Factors Associated With Awareness of Precision Medicine Among Individuals With Depression and Anxiety

**DOI:** 10.7759/cureus.62173

**Published:** 2024-06-11

**Authors:** Soji Ojo, Emmanuel O Ilori, Bernard Wiredu, Okelue E Okobi, Eziuche Kanu, Roseline Igbadumhe

**Affiliations:** 1 Public Health, University of Texas Health Science Center at Houston, Houston, USA; 2 Orthopedic Trauma, Texas Health Harris Methodist Hospital, Dallas, USA; 3 Psychiatry and Behavioral Sciences, Garnet Health Medical Center, Middletown, USA; 4 Internal Medicine/Oncology, St. James School of Medicine, Park Ridge, USA; 5 Family Medicine, Larkin Community Hospital Palm Springs Campus, Miami, USA; 6 Family Medicine, Medficient Health Systems, Laurel, USA; 7 Family Medicine, Lakeside Medical Center, Belle Glade, USA; 8 Department of Public Health, National Open University, Abuja, NGA; 9 Psychology, Alaska Native Medical Center, Anchorage, USA

**Keywords:** united states, education, health-related social media, sociodemographic factors, awareness, mental disorders, precision medicine

## Abstract

Objective: This study investigates the prevalence and determinants of awareness of precision medicine among a nationally representative sample of individuals with self-reported depression and anxiety in the United States."

Methods: Data were obtained from the Health Information National Trends Survey (HINTS) 5, Cycle 4, which is a study administered by the National Cancer Institute and is nationally representative. The survey, conducted between February and June 2020, targeted non-institutionalized, civilian US adults aged 18 years and older. Utilizing survey-weighted logistic regression, predictors of precision medicine awareness were assessed, encompassing sociodemographic, health-related, and technological factors.

Results: Among 890 individuals with self-reported depression and/or anxiety, approximately 15.3% reported awareness of precision medicine. Participants who had a higher level of education and those who had increased health-linked social media usage were three times more likely to be aware of precision medicine compared to those who did not. Old age was also positively associated with increased awareness.

Conclusion: The present study’s findings have disclosed an alarming lack of awareness of precision medicine, particularly among mentally ill persons with anxiety or depression, in which the targeted subgroups, including individuals with lower education levels and limited health-linked social media utilization, indicated lower levels of awareness. As such, it is recommended that such disparities be tackled using customized interventions along with educational initiatives, as this is likely to improve awareness levels while also ensuring equitable and increased access to precision medicine within the context of mental health.

## Introduction

Being an emergent field in medicine, precision medicine mainly refers to individualized and customized treatments aligned with personal attributes, definite healthcare requirements, and the genetic makeup of the patient [1]. This advanced approach to healthcare service provision has prompted an upsurge of assurance and optimism within the global medical community, providing the necessary pathway to achieving customized treatment interventions capable of altering the treatment landscape for different medical conditions [2]. Within the mental health context, in which psychiatric disorders like anxiety and depression have been acknowledged to considerably impact millions of individuals globally, there is profound potential for precision medicine to transform treatment standards.

Globally, psychiatric disorders account for approximately a third of disabilities and have been attributed to significant levels of individual and societal burden [3,4]. Even though existing pharmacotherapies and non-pharmacotherapies have assisted several patients, the rates of partial or no response, undesirable negative effects, and delayed effects have remained higher [5]. Further, current diagnostic criteria for mental health disorders are highly reliant on the presenting signs and symptoms and have failed to reflect the evidence from other behavioral and neurobiological systems, even as the lack of objective clinical measures has resulted in a wider variability in treatment responses and patient experiences [6,7]. Mental health disorders are among the leading causes of global health-associated burden, and depression and anxiety disorders are key contributors to the burden [3,4]. Moreover, it is estimated that 3.8% of the global population experiences depression, including 5% of adults and 5.7% of individuals aged 60 years and older [3-7]. Globally, nearly 280 million individuals have depression, and the disorder is approximately 50% more widespread among women than men [4-7]. Reassuringly, recent advances have ushered in a new understanding of the biological mechanisms underlying mental disorders. Thus, novel and innovative approaches to deliver personalized patient-tailored individual treatments have become necessary.

As precision medicine becomes increasingly integrated into health care prevention and treatment models, research into the awareness and patterns of precision medicine adoption among those with mental disorders must be undertaken. While there is considerable literature on the role of precision medicine in several clinical populations, such as those with cardiovascular diseases and cancer, there is relatively scant data regarding the prevalence and factors associated with precision medicine literacy in mental health contexts. For example, a survey on US adults' attitudes towards pharmacogenetic testing found that younger individuals, whites, those with a college degree, and individuals who had experienced side effects from medications were more inclined to participate in pharmacogenetic testing [8]. For instance, individuals aged 18-54 years were more likely to partake in pharmacogenetic testing (OR=1.25, p=0.012, 95% CI [0.73, 2.15]) in comparison to individuals aged 55 and above (OR=0.48, p=0.0005, 95% CI [0.278, 0.841]) [8]. Another large observational study involving more than 7000 US adults [9] showed that gender, race, educational attainment, and socioeconomic status were independently associated with greater awareness of genetic testing. While these studies offer some insights into the awareness of precision medicine approaches among the general population, less is known about the prevalence and factors associated with the awareness of precision medicine among individuals with psychiatric disorders.

This study aims to determine the prevalence of awareness and identify factors associated with awareness of precision medicine among individuals in the United States with self-reported depression and/or anxiety, using data from the Health Information National Trends Survey (HINTS) 5, Cycle 4. As such, the present study aims to provide essential insights and assist in informing the strategies and guidelines that will increase the adoption and approval of precision medicine by persons faced with the challenge of navigating psychiatric disorders.

## Materials and methods

Study setting, design, and sample

Data for this study were drawn from the National Cancer Institute's (NCI) Health Information National Trends Survey (HINTS) 5, Cycle 4 (H5C4), a nationally representative survey of the US adult population [10]. The HINTS collects information related to preventive health behaviors, the use of health information technology, and cancer control and prevention among non-institutionalized, civilian US adults (≥18 years). The HINTS 5 Cycle 4 study was mainly carried out between February 24 and June 24, 2020, through email, and the study sampling was based on the database of the Marketing Systems Group (MSG). The data was additionally stratified based on the minority population concentration and the addresses in census tracts with African Americans and Hispanics, who made up ≥ 34% of the overall study population, being allotted to the higher minority stratum. The remaining addresses were then allotted to the lower minority strata. To ensure the accuracy of the estimates for minority groups, an oversampling of the higher minority strata was performed [11].

Within the stratified sampling frame, the initial task involved the selection of addresses in every stratum through the use of the equal probability technique. This was followed by the selection of a single adult person from every household through the use of the Next Birthday respondent selection method. The Next Birthday respondent selection refers to a probability method that does not require that all household members be enumerated but rather requires the interviewer to speak and interview the household member whose birthday is next. Details regarding the study procedures and data collection process were reported elsewhere [10]. The Westat IRB approved the HINTS study, leading to the exemption from the US NIH Office of Human Subjects Research Protection. Additionally, a de-identification of the HINTS data was performed, and the data was made available publicly [12], in addition to our secondary data analysis being exempted from approval by the institutional review board. The present study has adhered to the STROBE reporting guidelines for cross-sectional observational studies [13]. Also, for the present study, data analysis was conducted in November 2023.

15,347 households received the questionnaires in H5C4; the response rate was 36.7%. Overall, 3,865 respondents were included in HINTS 5 cycle 4. For this study, individuals with depression and/or anxiety were ascertained by the survey question. Have you ever been told by a doctor or any health professional that you had an anxiety disorder or depression (yes/no)? “Yes” respondents were classified as having depression and/or anxiety [14]. The exclusion from the analyses entails all participants who failed to respond to this question, along with those whose data were 'missing’. The final analytic sample comprised 890 individuals who endorsed depression and/or anxiety diagnoses. Among the potential biases acknowledged was the selection bias, which is an error occurring from the identification of the study sample/population and is mainly a systematic error that is introduced in the course of screening/selection and facilitates the wrong conclusions in relation to the hypothesis under investigation. To tackle the potential selection bias, we utilized the play of chance in assigning participants to different groups, as this makes the process unpredictable and prevents systematic divergences between the groups. Consequently, the other potential bias noted in this study was the no-response bias, which refers to the failure to aptly secure all selected sample units' participation, particularly in population-based surveys. To handle the potential no-response bias, we set expectations with the study participants and clarified the survey objective and the time it would take. Additionally, the participants were reassured that they would participate anonymously and that all their personal information would be de-identified.

Outcome measure

The dichotomized variable that represented whether or not a study respondent was aware of precision medicine was the main outcome measure in our study. Awareness of precision medicine was determined based on responses of “yes” or “no” to the survey question, “Before completing this survey, had you ever heard of approaches like precision medicine?

Participant characteristics

Sociodemographic and health-related variables included age, sex, self-reported race/ethnicity, educational attainment, annual household income, access to a regular provider, rural/urban residence, the presence of comorbidities, and health-related social media usage. We hypothesized that awareness of precision medicine among individuals with self-reported depression and/or anxiety would vary by sociodemographic and health-related factors such as age, sex, self-reported race/ethnicity, educational attainment, annual household income, access to a regular provider, rural/urban residence, presence of comorbidities, and health-related social media usage. No variable selection was performed, given that such strategies normally result in higher false positives. As such, the covariates in our study were a priori determined based on the subject matter's understanding and knowledge.

To construct the health-related social media usage variable, answers to the research questions included: "During the previous 12 months, (i) Have you shared any health information on social media, like Twitter or Facebook? (ii) Have you taken part in any online forum or even support group for individuals with comparable medical and health issues? Lastly, (iii) Have you viewed any health-based videos on online platforms like YouTube?" were evaluated. For all the questions, the answer options were restricted to "yes" and "no," and, as a result, they were dichotomized. Subsequently, a composite variable for the categorization of health-linked social media use was developed. Participants who reported having one or more health-linked social media usages were then classified as social media users and were effectively coded as one and two; correspondingly, even the participants who reported no health-related use of social media were aptly classified as non-users and coded zero.

Statistical analysis

The analysis of the study sample entailed the adjustment of the HINTS' complex survey design. Overall, the survey-weighted prevalence of precision medicine awareness was approximated through various sociodemographic factors, including sex/gender, age, self-reported ethnicity, level of educational attainment, yearly household income, urban/rural residence, regular access to a healthcare provider, the existence of comorbidities, and health-associated use of social media, through the use of descriptive statistics such as chi-squared tests.

The outcome variable in our main analysis was dichotomized to represent being aware or not aware of precision medicine. A survey-weighted logistic regression was conducted to identify the factors associated with awareness of precision medicine. Since our outcome variable was dichotomous, the logistic regression method was appropriate, and to account for the complex survey design of HINTS, survey-weighted logistic regression was implemented. All the sociodemographic factors (i.e., age, sex, self-reported race/ethnicity, educational attainment, and annual household income), access to a regular provider, rural/urban residence, presence of comorbidities, and health-related social media usage served as covariates in the logistic model.

The statistical analyses were mainly completed through the use of the "svy" command of the Stata v.17.0 statistical software developed by StataCorp LP, based in College Station, Texas, USA. The eventual individual weights, along with the jack-knife replicate weights found in the H5C4 dataset, were subsequently employed in deriving the national-level approximates and the related standard errors. Every test was two-tailed, and even a p-value ≤ 0.05 was regarded as statistically significant.

## Results

Sample characteristics

A total of 890 participants were included in the final analytic sample and represented 60,796,771 persons aged 18 years and older who self-reported their experience of anxiety and depression, in addition to responding to survey items that enquired about their awareness with regard to precision medicine. Approximately 59.4% of the participants were aged between 18 and 49 years, even though 61.5% were female and 68.8% reported being non-Hispanic white. 64.2% reported having some college or higher educational attainment, in addition to 68.7% reporting having access to a regular healthcare provider. In general, nearly 15.3% of the participants who reported having depression/anxiety reported having an awareness of precision medicine.

Table 1 shows the demographic distribution of the study population. It also lists all examined covariates. Bivariate analysis indicates that participants with higher educational attainment, higher annual income, and increased health-related social media use reported greater awareness of precision medicine. Additionally, precision medicine awareness did not vary by ethnicity, gender, age, geographic residence, or the existence of medical comorbidity.

**Table 1:** Sociodemographic/health characteristics by awareness of precision medicine status among depressed/anxious: sample N = (890) (-): These cells were intentionally left blank

Demographic variables	Total (n=890), %	non-aware (n=734), %	Aware (n= 156), %	p-value
Gender	-	-	-	0.106
Female	61.5	86.9	13.1	-
Male	38.5	79.8	20.2	-
Age Group	-	-	-	0.649
18-34	28.1	82.5	17.5	-
35-49	31.3	85.8	14.2	-
50-64	26.5	88.3	11.7	-
65+	14.1	84.3	15.7	-
Education	-	-	-	0.001
Less than college	35.8	93.4	6.6	-
Some college/college/postgraduate	64.2	79.8	20.2	-
Household Income	-	-	-	0.008
	27.7	88.8	11.2	-
$20,000 to $34,999	10.7	93.2	6.8	-
$35,000 to $49,999	11.0	90.3	9.7	-
$50,000 to $74,999	15.1	89.0	11.0	-
$75,000 or more	35.5	75.6	24.4	-
Race	-	-	-	0.156
White	68.8	82.7	17.3	-
Black/African American	10.7	93.8	6.2	-
Hispanic	13.2	89.2	10.8	-
Others	7.3	79.9	20.1	-
Residence	-	-	-	0.578
Urban	87.5	85.1	14.9	
Rural	12.5	81.7	18.3	
Comorbidity	-	-	-	0.945
None	37.6	84.8	15.2	-
At least one comorbidity	62.4	84.5	15.5	-
Regular Provider	-	-	-	0.418
No	31.3	87.2	12.8	-
Yes	68.7	84.0	16.0	-
Health-related social media use	-	-	-	0.023
None	44.4	91.1	8.9	-
One form	34.0	80.6	19.4	-
Two or more forms	21.6	78.8	21.2	-

The multivariable regression analysis (Table 2) indicated that factors, including education level, age, and health-linked social media use, had significant correlations with greater odds of precision medicine awareness, particularly among persons with anxiety and/or depression. In particular, persons with some level of college education attainment (OR 3.29, 95% CI 1.38-7.87; p = 0.008) were highly liable to report awareness of precision medicine in comparison to individuals with less than college education attainment. Similarly, in comparison to the younger persons, older adults aged 65 years or more had higher odds with regard to clinical trial awareness (OR 2.85, 95% CI 1.04-8.08; p = 0.046). In addition, individuals who reported having two or more health-linked social media use were significantly more likely to endorse being aware of precision medicine in comparison to those who reported having no health-linked social media use (OR 3.17, 95% CI 1.39-7.19; p = 0.007).

**Table 2:** Multivariable logistic regression of predictors of awareness of precision medicine among depressed/anxious individuals: sample N = (890) C.I: Confidence interval, (-): Intentionally left blank

Demographic variables	Awareness of Precision Medicine, Adjusted Odds Ratio, 95% C. I	p-value
Gender	-	-
Female (Reference)	1.00	-
Male	2.35 (0.97, 5.70)	0.057
Age Group	-	-
18-34 (Reference)	1.00	-
35-49	0.92 (0.36, 2.36)	0.854
50-64	0.92 (0.38, 2.25)	0.851
65+	2.85 (1.03, 8.08)	0.046
Education	-	-
Less than college (Reference)	1.00	-
Some college/college/postgraduate	3.29 (1.38, 7.87)	0.008
Household Income	-	-
-	1.00	-
$20,000 to	0.60 (0.15, 2.40)	0.458
$35,000 to	0.81 (0.16, 4.03)	0.794
$50,000	0.88 (0.24, 3.19)	0.842
$75,000 or more	2.19 (0.83, 5.79)	0.112
Race	-	-
White (Reference)	1.00	-
Black/African American	0.69 (0.15, 3.15)	0.624
Hispanic	0.77 (0.15, 4.07)	0.757
Others	1.70 (0.53, 5.48)	0.365
Residence	-	-
Urban (Reference)	1.00	-
Rural	1.17 (0.40, 3.45)	0.768
Comorbidity	-	-
None (Reference)	1.00	-
At least one comorbidity	0.83 (0.38, 1.81)	0.626
Regular Provider	-	-
No (Reference)	1.00	-
Yes	1.16 (0.49, 2.71)	0.735
Health-related social media use	-	-
None (reference)	1.00	-
Only 1 form	1.57 (0.75, 3.28)	0.223
Two or more forms	3.17 (1.39, 7.19)	0.007

## Discussion

The objective of the present study was to assess the literature on precision medicine awareness in the context of mental health. To attain this objective, data drawn from the 2020 Health Information National Trends Survey was assessed to ascertain the awareness levels with regard to precision medicine as well as explore the extant correlations with the sociodemographic and health-related attributes in the nationally representative sample population with self-reported anxiety and/or depression. This study reveals a critically low awareness of precision medicine among individuals with self-reported depression and anxiety. We provide valuable insights into factors associated with precision medicine awareness in mental health contexts. Our results indicate that sexual age, educational attainment, and health-related social media usage are associated with increased awareness of precision medicine among adults with depression and/or anxiety.

Our study breaks new ground by exploring the landscape of precision medicine awareness within the realm of mental disorders, marking one of the initial investigations in this domain. The finding that only 15% of individuals in the US with depression and/or anxiety were cognizant of precision medicine represents an alarmingly deficient level of awareness. This percentage mirrors the strikingly low rates (23.8%) observed within a diverse cross-section of the US population, as reported in previous studies [15]. While no prior investigations specifically targeted awareness of precision medicine among those with mental illnesses, our findings concerning the remarkably low awareness levels among US adults experiencing depression and/or anxiety emphasize the urgent necessity for enhanced public health initiatives aimed at augmenting awareness of precision medicine in mental health contexts [14-16].

Within our study, sociodemographic factors revealed several significant insights. Notably, our analysis underscored a crucial link between age and awareness of precision medicine. One compelling explanation for this correlation might stem from the fact that older adults often contend with more medical complexities, a heightened susceptibility to conditions like cancer, and a greater need for preventative healthcare measures, such as vaccinations. Consequently, they tend to interact more frequently with the healthcare system, engaging in preventive screenings and treatment modalities. These increased healthcare encounters could potentially offer more occasions for healthcare professionals to introduce and discuss precision medicine methodologies, including genetic testing measures, thereby enhancing their familiarity with precision medicine concepts and bolstering their overall health literacy in this domain. This line of reasoning finds support in prior research. Studies, such as Onyeaka H et al.[14], have demonstrated a notable increase in genetic information awareness among older adults, as evidenced by a large sample of Japanese adults.

Our study results also indicate that educational attainment significantly influences awareness levels of precision medicine. Particularly, individuals with some college-level education attainment alongside those with some college-degree education were three times more likely to be aware of precision medicine compared to those with high school diplomas and lower levels of educational attainment. One plausible explanation for the association between higher education levels and higher awareness of precision medicine in this study is that higher education is associated with better health information-seeking behavior [17-19], allowing individuals to access accurate health information. Recently, large-scale national campaigns and awareness efforts have been instituted to improve public awareness and uptake of precision medicine in the US by leading health institutions in the US [20-22]. Yet the disparity in awareness of precision medicine due to the educational status observed in our study underscores the importance of targeted education and awareness campaigns among such vulnerable groups.

Our results suggest a role for social media in improving precision medicine uptake and adoption. We observed that respondents with anxiety and/or depression were three times more likely to report being aware of precision medicine if they utilized social media for health-associated functions compared to those who did not. This is consistent with the findings of earlier studies that suggested an increasingly significant role for social media in advancing health literacy across multiple clinical populations [23,24]. Thus, the analysis indicates that social media can be effectively used in the promotion of awareness with regard to precision medicine among mentally ill people.

Surprisingly, we observed no racial or socioeconomic disparities in awareness of precision medicine. Contradictory to the prevision studies [9,25], the findings of this study indicate that among individuals with anxiety and depression, there were no income or racial differences in precision medicine awareness. While differences in the sampled clinical population may account for our findings, another potential explanation could be that, given that the role of precision medicine in mental health care is still in its nascent phases, there may be limited differences in patient awareness across racial groups. However, as precision medicine becomes increasingly integrated into mental health care, future research must continue to explore any racial/socioeconomic disparities to ensure equitable access to and adoption of precision medicine in psychiatric populations.

Given the emerging role of precision medicine in managing mental disorders, the current study's findings offer valuable insights and provide directions for future studies on the determinants of precision medicine awareness and literacy among mental health populations. Our results also have substantial implications for informing policy guidelines to ensure equitable access and uptake of precision medicine in mental contexts.

Strengths and limitations

Our study has several strengths and limitations. For example, our study has utilized data drawn from the National Cancer Institute's (NCI) administered Health Information National Trends Survey HINTS 5, Cycle 4 (H5C4), which is a nationally representative survey of the US adult population, which makes the findings of the study generalizable and widely applicable. Still, the other notable strength of our study regards the study design. Thus, the survey research design used is an increasingly reliable inquiry method, given that the survey questions were standardized, as the questions are the same and phrased in precisely the same way. Further, our data is novel and extends the literature on precision medicine awareness among US adults with mental disorders (specifically depression and anxiety), which makes the findings up-to-date and more reliable. Nevertheless, our study has several limitations worth highlighting. First, the study population was limited to individuals with self-reported depression and/or anxiety, not encompassing all psychiatric disorders. Thus, these findings may not be generalizable to other psychiatric populations, such as those with serious mental illnesses. Second, survey data was collected using self-reported measures subject to recall bias. Third, the cross-sectional nature of the study design prevents us from drawing causal relationships. Future longitudinal studies are needed to explore the temporal relationship between education, age, health-related social media usage, and awareness of precision medicine. Fourth, due to the nature of the survey items, we could not assess other theoretical factors that may be associated with knowledge of precision medicine. This is because the survey questions are standardized, which makes it difficult for the interviewer to investigate anything apart from the general questions that can be understood by a broader range of individuals. Lastly, the HINTS is prone to low response bias, but robust statistical methods utilizing replicate weights have been applied to account for this. 

## Conclusions

Precision medicine has become a revolutionary and important tool in the advancement of personalized and customized treatments, as well as in enhancing health outcomes for mentally ill people. Depression and anxiety disorders have been associated with considerable socio-economic burdens, most of which are linked to the present diagnosis and treatment guidelines. However, at present, the approaches that have been adopted in both drug/treatment discovery and drug development for mental disorders, particularly depression and anxiety disorders, have been comparatively unsuccessful. As such, precision medicine seeks to customize mental healthcare more closely to the individual patient's needs and, in instances where it is informed by neuroscience, provides the opportunity to enhance accuracy with regard to disorder classification, disorder diagnosis, treatment decisions, and prevention interventions. Our study findings indicate that only one in six adults with anxiety and/or depression in the United States are aware of precision medicine. Further, the study findings indicate that a number of the patients' sub-groups, particularly those of younger age, lower educational attainment, and those with low social media usage for health-related purposes, may lack awareness of precision medicine. These findings are valuable and identify subgroups of people with mental disorders that may benefit from targeted interventions to improve awareness and ensure equitable access and adoption of precision medicine. The findings also highlight the need for a collaborative approach from several disciplines to pave the way for precision medicine/psychiatry to enhance prognosis and the quality of life for patients with depression and anxiety.
